# Novel Proteins for Future Foods: Current Status, Challenges, and Perspectives

**DOI:** 10.3390/foods14050862

**Published:** 2025-03-03

**Authors:** Malik Altaf Hussain, Li Li

**Affiliations:** School of Science, Western Sydney University, Richmond, NSW 2753, Australia; l.li7@westernsydney.edu.au

## 1. Introduction

Novel or alternative protein sources are a mega food innovation trend in the global commercial food sector. These are protein-rich ingredients sourced from plants, insects, fungi, algae, or animal cells, offering promising opportunities to design future foods. The current revolution in novel proteins is driven by several factors, including increased health and climate change consumer concerns. One of the strongest arguments for the consumption of protein-dense new non-animal foods is that they have a lower environmental impact than conventional livestock products [1].

Predicted global alternative protein market value could exceed USD 190 billion by 2028 [2]. Novel protein sources are getting unprecedented attention for future food security to develop sustainable and resilient food systems [3], reduce environmental impact, and provide nutritious food options for a growing global population (expected to reach 10 billion by 2050). As for now, the challenge is designing food systems to meet the growing demand for adequate, nutritious, safe, and sustainable foods. The alternative protein industry is crucial and has the potential to meet this demand through continued research, innovation, and investment in this sector [4]. Therefore, embracing new and alternative protein sources can help address the challenges associated with traditional protein production and contribute to a more secure and sustainable food future.

## 2. Current Status and Perspectives

Plant-based proteins, such as legumes and pulses, offer great versatility and wider acceptability for future food products because they are traditional and local protein sources for many nations worldwide [5]. Some reports indicated an increase of 302% in new packaged consumer goods, launched with a plant-based claim, between 2018 and 2022 [6]. A many-fold increase is anticipated in the use of forage-based domestic protein sources in the coming years. A transition of the protein industry towards more sustainable protein sources, such as plant-based substitutes, is vital. A combination of factors, including the demand for healthier foods, environmental concerns, and the need to ensure food security, is bringing a rapid shift in alternative protein acceptability. Government support for plant-based proteins and increased interest in investing in the sector have been seen in many countries. For example, the New Zealand Government announced an investment of more than USD 450,000 in a project worth around USD 890,000 initiated by Kernohan Engineering, NewFish, and Cawthron Institute in 2023 [7].

Emerging novel protein sources, together with plant-based proteins, appear to vary in health and/or nutritional functions, and unsurprisingly, the approaches to utilize them also differ [8]. For example, hydrolysates of insect proteins seem to be the focus of utilization exploration, while the entire collection of algal cells cultivated is often added to enhance product quality, as highlighted by Jain et al. [8]. The efforts to replicate traditional meat products with much reduced environmental cost have led to the development of cultured muscle cells, sometimes in algal cell medium for further reduction of greenhouse gas emissions, and at other times cultivating insect-derived cells instead of animal cells to minimize ethical concerns.

Novel proteins also bring significant opportunities for the growth of global food markets. Economic indicators positively demonstrate the alternative protein market growing across all regions. For example, the expected annual growth rate (CAGR) from 2021 to 2027 for the alternative protein markets is 18.5% and more than 17.5% in the Asia–Pacific region and the United States, respectively [9]. It is anticipated that plant-based protein will have the largest share in the future based on the trend noted in the United States where the plant-based market was more than USD 35 billion (out of USD > 50 billion alternative protein market) in 2020 [9]. Future economic growth and business opportunities are developing strategic collaborations and investments among major market players. This would help them not only in enhancing product reach but also in diversifying portfolios. Food companies and startups are focused on developing innovative and value-added products through active research and development to sustain market growth.

## 3. Challenges

With this rapid shift in consumption patterns, it is a crucial and challenging task to manage the food safety of alternative protein sources to protect consumer health, build trust in emerging food technologies, and ensure the long-term success and acceptance of these innovative products within the food supply chain. Singh [7] highlighted four major challenges that need to be overcome for the successful adoption of alternative protein as future foods:

1. Consumer acceptance and perception challenges

Alternative protein and food products containing novel protein sources face several challenges related to consumer acceptability and perception such as dietary preferences, unacceptable sensory attributes (the taste and texture), and a lack of awareness about these products’ environmental impacts and health benefits. For example, a general trend reported for insect-based food is that consumer acceptability remains a bottleneck among many populations, particularly in Western countries. Food neophobia appears to be the most commonly cited cause for concern [10,11], and using insects as livestock feed may receive higher acceptance [12].

2. Challenges related to food safety and risk of allergenicity

The occurrence of natural toxicants, chemical or microbiological contamination, growth of pathogenic microorganisms in the products, and allergen management are major food safety challenges. A recent contamination incident occurred when a few plant-based beverage products were recalled in July 2024 due to *Listeria monocytogenes* contamination and associated infection reported by the Canadian Food Inspection Agency [13]. It is well known that insects and insect-derived products can lead to a myriad of allergic reactions in sensitive people, such as those allergic to crustacean products [10,11,12]. Ongoing research efforts are essential to understand the allergenicity of new and modified protein sources to protect consumer health.

3. Technological challenges to scale up and production efficiencies

Processing technologies and scientific advancements are still in the developmental phase for many products using novel protein sources. Sophisticated biotechnology and substantial investment are needed to scale up the production of cell-based meats and fermentation-derived proteins at the commercial level. In the former case, for example, an immediate need is warranted to invest in and establish a much more affordable commercial-scale infrastructure for the production of cell cultivation media components, while technical breakthroughs in bioreactor design and cell growth to further lower production costs and improve product quality are being developed [8,14,15].

4. Regulatory hurdles and harmonization of food standards

International food standards lack harmonization in novel food standards, and significant regulatory disparities exist between regions at this stage [16]. The regulation of insect-based proteins is a typical example where regulatory hurdles require urgent attention. Developing universal, comprehensive, and standardized regulation could be an important step to harnessing the potential of insect-based proteins. Furthermore, genetically modified (GM) foods offer promising potential for addressing some of the greatest challenges of the 21st century such as food security and environmental conservation [17]. Like all new technologies, GM foods could also pose known and unknown risks to the consumer, including novel toxins, allergens, and reduced nutritional value. Many global food regulatory agencies have developed rigorous processes for assessing the safety of GM foods [18,19]; however, differences in the risk assessment approach and approval systems vary by country. For instance, the Japanese regulations do not distinguish between genetically modified plants, animals, and microorganisms, but the rules apply to different types of uses, such as cultivation, food use, and feed use [20].

Alternative proteins would play a crucial role in feeding the growing populations more sustainably than animal agriculture. They have great potential to shape the future global food system by making it more resilient to climate change and supply chain disruptions. However, steady progress toward a sustainable and resilient food system requires joint efforts from all stakeholders, including food safety authorities, industry leaders, and academic researchers, to address most of the abovementioned challenges.
